# A sequential explanatory mixed-methods study on costs incurred by patients with tuberculosis comorbid with diabetes in Bhavnagar, western India

**DOI:** 10.1038/s41598-023-27494-7

**Published:** 2023-01-04

**Authors:** Mihir P. Rupani, Sheetal Vyas

**Affiliations:** 1 Department of Community Medicine, Government Medical College Bhavnagar (Maharaja Krishnakumarsinhji Bhavnagar University), Near ST Bus Stand, Jail Road, Bhavnagar, Gujarat 364001 India; 2 Gujarat University, Ahmedabad, Gujarat 380009 India; 3 Department of Community Medicine, AMC-MET Medical College, Maninagar, Ahmedabad, Gujarat 380008 India; 4 Present Address: Clinical Epidemiology, Division of Health Sciences, ICMR - National Institute of Occupational Health (NIOH), Indian Council of Medical Research, Meghaninagar, near Raksha Shakti University, Ahmedabad, Gujarat 380016 India

**Keywords:** Tuberculosis, Health care economics, Health policy, Health services

## Abstract

Diabetes is one of the commonest morbidity among patients with tuberculosis (TB). We conducted this study to estimate the costs incurred by patients with TB comorbid with diabetes and to explore the perspectives of program managers as well as patients on the reasons and solutions for the costs incurred due to TB-diabetes. We conducted a descriptive cross-sectional study to estimate costs among 304 patients with TB-diabetes comorbidity registered in the public health system during 2017–2020 in the Bhavnagar region of western India, which was followed by in-depth interviews among program functionaries and patients to explore solutions for reducing the costs. Costs, when exceeded 20% of annual household income, were defined as catastrophic as this cut-off was most significantly related to adverse TB outcomes. Among the 304 patients with TB-diabetes comorbidity, 72% were male and the median (interquartile IQR) monthly family income was Indian rupees (INR) 9000 (8000–11,000) [~ US$ 132 (118–162)]. The median (IQR) total costs due to combined TB-diabetes were INR 1314 (788–3170) [~ US$ 19 (12–47)], while that due to TB were INR 618 (378–1933) [~ US$ 9 (6–28)]. Catastrophic costs due to TB were 4%, which increased to 5% on adding the costs due to diabetes. Health system strengthening, an increase in cash assistance, and other benefits such as a nutritious food kit were suggested for reducing the costs incurred. We conclude that, in addition to a marginal increase in the percentage of catastrophic costs, co-existent diabetes nearly doubled the median total costs incurred among patients with TB. Strengthening the TB-diabetes bi-directional activities, tailoring the cash transfer scheme for comorbid patients, and making the common two-drug combination diabetes tablets available at government drug stores would help TB-diabetes comorbid patients cope with the costs of care.

## Introduction

India has been in focus on the world scenario due to tuberculosis (TB). The country, reporting the highest number of estimated incident cases of TB (1.5 million out of an estimated 5.8 million global cases in the year 2020)^1^, has set an ambitious target of eliminating TB by the year 2025 as against the conventional deadline of 2030 for the same^2^. India is also estimated to report the second-highest number of cases of diabetes (74 million out of an estimated 537 million global cases in the year 2021)^3^. Care for diabetes, important comorbidity among patients with TB, has been emphasized to be addressed through collaborative activities with TB care as early detection is likely to reduce the costs incurred by the patients^4,5^.

As per the End-TB strategy, by the year 2030, families with a patient with TB should incur zero catastrophic costs—an indicator suggesting the tendency of a family to be pushed below the poverty line due to care for TB^6^. The prevalence of catastrophic costs due to TB has been estimated to be 43% globally^7^, whereas it is ranging from 4 to 68% in India^8,9^. Among patients with TB-diabetes comorbidity in Kyrgyzstan, it was reported that the costs incurred due to drugs for diabetes were the most prominent^10^. Another study from the same group of authors found patients with comorbid TB-diabetes as more likely to incur catastrophic costs when compared with patients with TB^11^.

Diabetes increases the risk of developing active TB, while TB has been reported to alter glycemic control among patients with diabetes^12^. The risk of acquiring TB increases among the poor, and TB worsens poverty by afflicting the working population^13,14^. Further, even with the provision of free care, TB-affected households employ dissaving to offset the costs of care^8^. The costs incurred for the care of diabetes were observed to be higher than TB care due to the chronic nature of the disease requiring lengthier follow-up^11^. Evidence on costs incurred due to the dual existence of TB-diabetes is scarce. We hypothesize that costs incurred due to diabetes might significantly increase the economic burden among patients with TB-diabetes, perhaps necessitating initiatives aimed at comorbid TB patients. We conducted this study to estimate the costs incurred by patients with TB comorbid with diabetes during TB treatment at a public health facility in the Bhavnagar region of western India. We also explored the perspectives of program managers as well as patients on reasons and solutions for the costs incurred due to TB-diabetes.

## Methods

### Study design and duration

We conducted a mixed-methods research among patients with tuberculosis comorbid with diabetes in the Bhavnagar region of Gujarat state. A cross-sectional descriptive study for estimating costs incurred by patients with TB comorbid with diabetes was followed by in-depth interviews among the patients as well as program functionaries for exploring reasons and solutions for the increased costs. The theoretical underpinning of the qualitative component was the constructivist paradigm and we planned to describe the codes/categories for the reasons and solutions for increased costs among TB-diabetes comorbid patients (descriptive design). The current study's methodological emphasis was phenomenology, and in-depth interviews were undertaken to explore the phenomenon experienced by the study participants. The cross-sectional study was conducted from July 2019 to December 2020, the in-depth interviews were conducted in January–February 2021.

### Study setting

Bhavnagar, a sparsely populated region located in Gujarat state (western part of India), is administratively divided into a district (largely countryside) as well as a city (semi-urban conglomerate). Bhavnagar district has a population of ~ 0.28 million, whereas the city has a population of ~ 0.06 million. The Bhavnagar region reports approximately 3000 incident cases of TB every year^15^. A decentralized model of service delivery is employed for TB through a network of government health facilities, private practitioners, and not-for-profit health facilities. The majority of primary health centers (PHCs) are designated microscopy centers, the majority of community health centers (CHCs) have X-ray facilities, and sub-district/district hospitals execute sophisticated diagnostic procedures such as rapid molecular testing, and culture-sensitivity^16^. Bhavnagar district has 10 administrative TB units (TUs), comprising 62 public health institutions (PHIs), including 48 PHCs, 10 CHCs, two sub-district hospitals, one district TB center, and one medical college-attached advance TB diagnostic center. Bhavnagar city is divided into two administrative TB units (TUs), comprising 13 urban PHCs.

### TB and diabetes programs and the collaborative framework

The national TB program in India has been recently rechristened as National TB Elimination Program (NTEP) to eliminate the disease by the year 2025^2^. The delivery of services under NTEP is through a network of public health institutions (PHIs), district TB centers, and tertiary care hospitals^16^. The National Program for Prevention and Control of Cancer, Diabetes, Cardiovascular Diseases and Stroke (NPCDCS) is an umbrella program for non-communicable diseases in India, the service delivery being similar to that of NTEP^17^. The NPCDCS offers a range of services, from health promotion at sub-health centers to rehabilitation and palliative care at tertiary care hospitals^17^. Diabetes is generally treated through public health facilities, which provide basic diagnostics and common medicines^17^. However, in India, the private sector (general practitioners, alternative systems of medicine, specialists) plays a predominant role in the healthcare service industry through individual clinics as well as corporate hospitals. Recognizing diabetes as important comorbidity among patients with TB and due to the bi-directional causality, the national framework for joint TB-diabetes collaborative activities was launched in India^5^. The collaborative framework entails bi-directional screening and management for patients with either of the two diseases.

### Cash assistance for patients with TB

A cash transfer program of credit of 500 Indian rupees (~ US$ 7) per month directly to the bank account of patients with TB was launched to facilitate the purchase of nutritious food during the course of treatment (irrespective of the duration of treatment)^18^. In addition, patients belonging to socially/ economically deprived castes were entitled to an additional credit of the same amount per month for a period of 6 months through a social welfare scheme.

### Study population

#### Quantitative

We included all adult patients with TB comorbid with diabetes, registered in the public health system between January 2017 and December 2020 in the Bhavnagar region of Gujarat state. We excluded re-treated patients, who had human immunodeficiency virus (HIV), or who refused to give written informed consent to participate in the study.

#### Qualitative

We included 17 program functionaries and eight patients with TB comorbid with diabetes for the in-depth interviews for exploring their perspectives on reasons and solutions for increased costs. The program functionaries included nine senior treatment supervisors, six TB health visitors, a district program coordinator, and a district TB officer—all working in the Bhavnagar region. The saturation of information was the criterion for discontinuing additional interviews. Important points were jotted down at the end of each interview and compared with successive interviews to determine saturation. None of the study participants refused to participate in the study. The interviews with program officials lasted around 25–40 min, whereas the interviews with patients lasted about 15 min.

### Sampling, recruitment, and data collection

#### Quantitative

The list of patients registered in the public health system between January 2017 and December 2020 was accessed from Nikshay (an online portal of registered patients with TB). The list, in the form of a Microsoft Excel sheet, was filtered to meet our eligibility criteria—patients < 18 years of age, retreated patients, or patients without diabetes were removed. With the help of TB program functionaries, eligible patients were traced using their addresses from Nikshay. A written informed consent procedure was conducted at their homes, which was followed by the data collection. The questionnaire for data collection included socio-demographic information, information on TB treatment, a cost survey^19^, questions on assets owned, and any negative coping strategy employed by the family. The treatment outcomes of the patients were obtained from the Nikshay portal.

#### Qualitative

The interview guide was designed to elicit perspectives on the reasons and solutions for the high costs of TB and diabetes care. The in-depth interviews were conducted at a time and place convenient for the patients as well as program functionaries. The sampling was purposive—those who were more knowledgeable or likely to respond were selected. Some of the patient's family members were present during the interviews that were conducted at their residences.

The first author conducted all of the interviews in Gujarati (the native language) and audio-recorded them. Both authors have an MD in Community Medicine and have undergone training in qualitative research. At the time of the study, both authors, one male and one female, were working as professors in medical colleges. Before the research started, the first author was acquainted with the majority of the program functionaries. The study participants were aware of the author's past work as well as the potential benefits of the research findings.

### Definitions (quantitative)

#### Costs

Costs were categorized as direct medical, direct non-medical, and indirect costs^8,19^. Direct medical costs were costs incurred due to hospitalization (day charges), consultation, charges for radiology, laboratory investigations or other procedures, medicines, and any nutritional supplements^8,19^. Direct non-medical costs were costs incurred due to travel, food, accommodation of patient and any accompanying member, and opportunity costs (costs paid for any household chores or due to any work outsourced in absence of patient/ accompanying member at home) during clinic visits^8,19^. Indirect costs were the sum of wage loss of the patient, wage loss of accompanying members, and income loss of the family (family income before TB minus family income after TB)^8,19^. The total costs were the sum of all the aforementioned costs (any amount received through cash assistance or reimbursement was subtracted)^8,19^. During the period of data collection, the average exchange rate of one US dollar was equal to 68 Indian rupees for the years 2017–2020.

#### Combined TB-diabetes costs

Costs incurred by patients due to TB was calculated from the onset of symptoms of TB till treatment completion. For study participants on treatment for TB, the cost survey covered all costs up until the time of enrolment in the research. We then made phone calls, depending on as to where the patient was in the treatment phase, to collect data on costs incurred till treatment was completed. Up to three phone calls were made to obtain the information on costs incurred due to adverse drug reactions, medicine collection visits, and any follow-up sputum tests. Costs incurred due to diabetes were calculated as costs from the time of diagnosis and treatment at the first detection till the time of completion of treatment of TB (that is, costs of diabetes were estimated for the duration of TB treatment). Combined TB-diabetes costs were the sum of costs incurred due to TB and diabetes.

#### Catastrophic costs

Catastrophic costs were defined when the total costs incurred due to TB exceeded 20% of the annual household income of the family^8,19^. For comparison, we also calculated catastrophic costs incurred due to combined TB-diabetes. We calculated annual household income by multiplying the TB-affected family’s self-reported monthly income by 12.

#### Standard of living (SLI) index

SLI index was calculated from information on the ownership of different assets by the families. A summary score ranging from 1 to 23 was calculated, with scores 1–7 defined as low SLI and 8–23 defined as middle/ high SLI^8,20^.

#### Treatment outcomes

Treatment success was defined when patients with TB, at the end of treatment, are either cured (smear or culture-negative) or have completed treatment (without evidence of failure or clinical deterioration)^16^. Treatment outcomes were defined as unfavorable when a patient with TB tested positive by smear or culture at the end of treatment (failure), interrupted treatment for one month (lost to follow-up), or, died while on treatment^16^.

### Analysis

#### Quantitative

Costs were estimated using the Statistical Package for Social Sciences (SPSS) software version 23 and expressed as median (interquartile range IQR) in Indian rupees (INR).

#### Qualitative

The audio recordings of in-depth interviews were transcribed from Gujarati into English and typed into a Microsoft Word document. The transcripts were not shared with the participants for feedback or correction. Both investigators assigned codes to the transcript simultaneously with consensus using the ‘comment’ tool of Microsoft Word. Codes were entered in a Microsoft Excel sheet for grouping them as categories under two pre-defined themes—reasons and solutions for increased costs. However, the assignment of the codes was inductive in nature as it was led by our study’s data. The results were not presented to the participants for comments. We used the framework method to describe the categories in a tabular form^21^.

### Ethics approval and consent to participate

Approval was obtained from the Institutional Review Board (Human Ethics Committee) of Government Medical College Bhavnagar (no. 868/2019, dated 08-07-2019). Written informed consent for participating in the study was obtained from the study participants. The study was performed in accordance with the Declaration of Helsinki.

## Results

### Quantitative

#### Characteristics of patients

**Figure 1:**
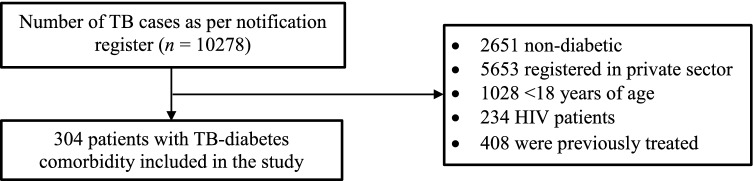
Selection process of patients registered in the public health system with tuberculosis comorbid with diabetes from January 2017 to December 2020 in Bhavnagar.

We included 304 patients with TB comorbid with diabetes meeting our eligibility criteria (Fig. 1, the response rate was 100%). The median (IQR) age of 304 TB-diabetes comorbid patients was 52 (42–61) years, 72% were male, 80% had no formal education, and 64% were residing in urban areas (Table 1). Among the 304 patients, the median (IQR) monthly family income was Indian rupees 9000 (8000–11,000) [~ US$ 132 (118–162)], 12% had a low SLI index, and 10% were sole earners in their family. Fourteen percent of the 304 patients had an unfavorable treatment outcome, 19% and 11% of the patients had their first TB and diabetes visit at a private provider respectively, and 8% and 4% were hospitalized during their first TB and diabetes visit respectively.

**Table 1:** Characteristics of patients with TB comorbid with diabetes from January 2017 to December 2020 in Bhavnagar (n = 304).

Characteristic	Number (%) or median (IQR)
**Socio-demographic characteristics**	
Age in years	52 (42–61)
Male	220 (72)
**Educational status**	
No formal education	242 (80)
Primary (7th pass)	43 (14)
Secondary (10th pass) and above	19 (6)
Married	281 (92)
Scheduled caste (SC)/scheduled tribe (ST)	22 (7)
Extended family (vs nuclear family)	266 (87)
Urban residence	195 (64)
Current tobacco smoking	81 (27)
Current regular alcohol consumption	5 (2)
**Economic characteristics**	
Cash assistance received for TB (n = 190)	152 (80)
Amount received in INR (n = 190)	3000 (750–3000) [~ US$ 44 (11–44)]
Monthly family income in INR	9000 (8000–11,000) [~ US$ 132 (118–162)]
Below poverty line (BPL) card	103 (34)
**Standard of living (SLI) index**	
Low (SLI score 1–7)	36 (12)
Middle/high (SLI score 8–23)	268 (88)
Employed in paid work before TB diagnosis	116 (38)
Currently in paid work	97 (32)
Sole earner in the family	30 (10)
**Clinical characteristics—TB**	
**Sputum acid-fast bacillus smear grade**	
Negative	107 (35)
Scanty	55 (18)
1+	77 (25)
2+	37 (12)
3+	28 (9)
Extrapulmonary TB	33 (11)
Drug-resistant TB	6 (2)
**Phases of treatment**	
Intensive phase of TB treatment	41 (13)
Continuation phase of TB treatment	42 (14)
Treatment completed	221 (73)
First TB visit with a private provider	57 (19)
Hospitalized due to TB at the first visit	25 (8)
**Treatment outcomes**	
Successful treatment outcomes	
Treatment completed	61 (20)
Cured	201 (66)
Unfavorable treatment outcomes	
Death while on treatment	30 (10)
Lost to follow up	6 (2)
Treatment failure	6 (2)
**Clinical characteristics—diabetes**	
First diabetes visit with a private provider	34 (11)
Hospitalized due to diabetes at the first visit	13 (4)

#### Costs due to TB, diabetes, and combined costs

Due to TB, the direct medical costs were Indian rupees (INR) 0 (0–0), direct non-medical costs were INR 210 (120–330) [~ US$ 3 (2–5)], and indirect costs were INR 354 (200–813) [~ US$ 5 (3–12)] (Table 2). When compared with diabetes, the indirect costs incurred due to TB were higher, while the direct non-medical costs due to TB were lower. Due to TB-diabetes, the combined direct medical costs were INR 0 (0–0), combined direct non-medical costs were INR 590 (360–998) [~ US$ 9 (5–15)], and combined indirect costs were INR 595 (339–1046) [~ US$ 9 (5–15)]. Among the direct non-medical costs, travel to attend the health facilities was the most prominent, while the wage loss of accompanying members mainly contributed to the indirect costs. The median (IQR) total costs due to combined TB-diabetes were INR 1314 (788–3170) [~ US$ 19 (12–47)], while that due to TB were INR 618 (378–1933) [~ US$ 9 (6–28)].

**Table 2:** Median (IQR) costs in Indian rupees (INR) incurred by patients with TB comorbid with diabetes from January 2017 to December 2020 in Bhavnagar (n = 304).

Categories of costs	TB costs	Diabetes costs	Combined TB-diabetes costs
Direct medical	0 (0–0)	0 (0–0)	0 (0–0)
Day charges	0 (0–0)	0 (0–0)	0 (0–0)
Consultation	0 (0–0)	0 (0–0)	0 (0–0)
Radiography	0 (0–0)	0 (0–0)	0 (0–0)
Laboratory	0 (0–0)	0 (0–0)	0 (0–0)
Procedures	0 (0–0)	0 (0–0)	0 (0–0)
Drug	0 (0–0)	0 (0–0)	0 (0–0)
Prescribed nutrition	0 (0–0)	0 (0–0)	0 (0–0)
Direct non-medical	210 (120–330) [~ US$ 3 (2–5)]	340 (180–600) [~ US$ 5 (3–9)]	590 (360–998) [~ US$ 9 (5–15)]
Travel to attend health facility	200 (120–320) [~ US$ 3 (2–5)]	320 (180–580) [~ US$ 5 (3–9)]	570 (360–920) [~ US$ 8 (5–14)]
Food purchased to attend health facility	0 (0–0)	0 (0–0)	0 (0–0)
Accommodation to attend health facility	0 (0–0)	0 (0–0)	0 (0–0)
Opportunity costs during clinic visits	0 (0–0)	0 (0–0)	0 (0–0)
Indirect	354 (200–813) [~ US$ 5 (3–12)]	200 (100–400) [~ US$ 3 (1.5–6)]	595 (339–1046) [~ US$ 9 (5–15)]
Loss of wages to attend health facility	100 (0–400) [~ US$ 1.5 (0–6)]	0 (0–200) [~ US$ 0 (0–3)]	113 (0–622) [~ US$ 2 (0–9)]
Wage loss of accompanying member	200 (100–300) [~ US$ 3 (1.5–4)]	100 (0–200) [~ US$ 1.5 (0–3)]	400 (100–550) [~ US$ 6 (1.5–8)]
Household income loss due to TB	0 (0–0)	–	–
Total costs	618 (378–1933) [~ US$ 9 (6–28)]	595 (380–1080) [~ US$ 9 (6–16)]	1314 (788–3170) [~ US$ 19 (12–47)]

Among the 304 patients, 58 (19%), 30 (10%), and 64 (21%), respectively, incurred > 0 direct medical costs due to TB, diabetes, and combined TB-diabetes (Supplementary Table S1). The median (IQR) direct medical cost for the 30 patients who incurred > 0 costs due to diabetes was INR 1050 (295–3725) [US$ 15 (4–55)] (Supplementary Table S1). Furthermore, patients who had their first consultation at a private health facility were more likely to incur direct medical costs (Supplementary Table S2).

#### Catastrophic costs and coping

At the 20% cut-off of annual household income, the percentage (95% CI) of households facing catastrophic costs due to TB was 4% (3–7%), which marginally increased to 5% (3–8%) on adding the costs due to diabetes (Supplementary Table S3). The median (IQR) total costs for the 4% of patients who incurred TB catastrophic costs was INR 26,026 (13,955–50,143) [US$ 383 (205–737)], whereas the median (IQR) annual household income was INR 8000 (1655–11,500) [US$ 118 (24–169)]. Among the 304 patients, 5% of the patients used at least one coping strategy to overcome the costs incurred (Supplementary Table S4). Two percent of the patients borrowed money as a loan, 3% lost employment after TB diagnosis, and the median (IQR) days of work lost due to TB was 45 (30–60).

### Qualitative

**Figure 2:**
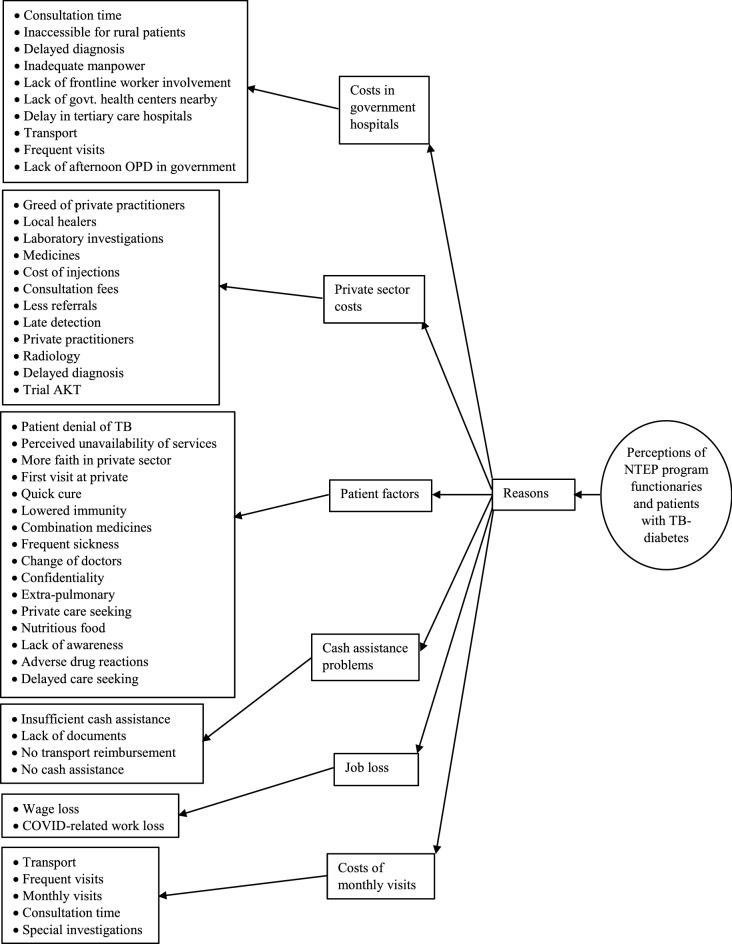
Reasons for increased costs due to TB and diabetes as perceived by program functionaries and patients in Bhavnagar.

**Figure 3:**
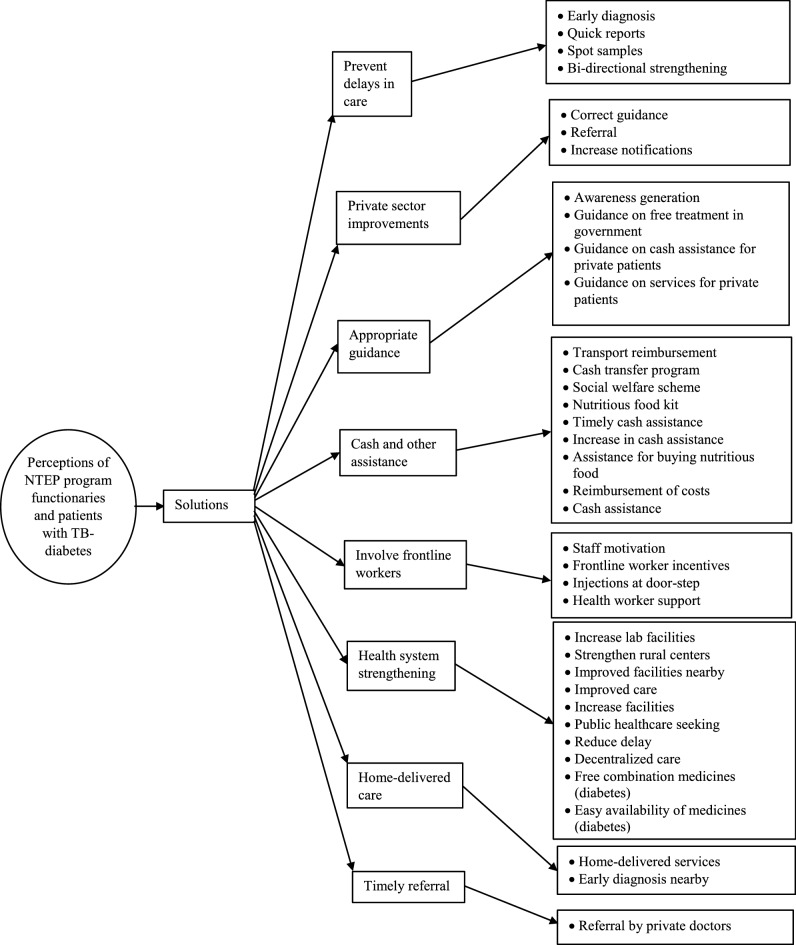
Solutions for increased costs due to TB and diabetes as perceived by program functionaries and patients in Bhavnagar.

Among the 17 program functionaries, the median (IQR) work experience was 7 (5–20) years and 3 were female. Among the 8 patients with TB comorbid with diabetes, the median (IQR) age was 50 (43–62) years and 4 were male. The analysis of the transcript brought forth various codes (under different categories) for reasons (Fig. 2) and solutions (Fig. 3) for increased costs due to TB and diabetes.

#### Reasons for increased costs of TB-diabetes

Costs incurred for clinic visits to government/ private hospitals, certain patient factors, and problems in availing cash assistance were the reasons perceived for increased costs due to TB/ diabetes (Table 3). Costs incurred for monthly follow-up visits were unique to diabetes, whereas costs incurred due to loss of job were unique to TB (refer to Supplementary Table S5 for a description of each code).

**Table 3:** Framework analysis describing categories of reasons for increased costs incurred due to TB and diabetes in Bhavnagar.

Categories of reasons for increased costs	Program functionaries’ perception		Patients’ perception	
	TB	Diabetes	TB	Diabetes
Costs in govt. hospitals	√		√	
Private sector costs	√	√	√	√
Patient factors	√	√	√	√
Cash assistance problems	√	√	√	
Job loss	√		√	
Cost of monthly visits		√		√

##### Costs in govt. hospitals

Inaccessibility for rural patients, lack of govt. health centers nearby, delays in tertiary care hospitals, lack of frontline worker involvement, inadequate manpower, transport fare, and requirement of frequent visits were some of the reasons perceived for costs incurred by patients with TB-diabetes at govt. hospitals.

*If the patients are from the city area, then they have to incur fewer costs, but, from the rural areas, they have to arrange a special rickshaw. They do not have any health center with diagnostic facilities, so they have to necessarily come here [tertiary care hospital] which increases their costs*. (TB health visitor, 6 years of experience)

*Patients have to come every month or once in two months depending on how the treating doctor asks to come for follow-up. Doctors have to see that after detection, how the patients are maintaining their blood sugar levels. So, at first detection, patients will be asked to come within a week to see the effect of the anti-diabetic drugs. In the first month, they might have to do two visits, but then once the patient is stable, then monthly or 1.5 monthly follow-ups will be done.* (District TB officer, 20 years of experience)

*There is no physician in a rural area, no MBBS doctor. We do not have X-ray facilities. We refer them to higher centers for X-ray.* (Senior treatment supervisor, 20 years of experience)

##### Private sector costs

The greed of private practitioners, consultation fees, local healers, expenses of laboratory investigations and medicines, and the cost of injections were some of the reasons for incurring costs in the private sector. Patients felt that the private practitioners continue to treat them till they can pay, instead of referring them timely to the govt. health facilities.

*Private doctors see patients according to their paying capacity. Once the doctor feels that the patient will no longer be able to purchase the medicines as per his requirement, he refers the patient to another doctor. He becomes an advocate, instead of a doctor.* (66 years, male patient)

*For diabetes, every month, I have to spend 400* [Indian rupees] *for the report, 200 for consultation, and 1700 for medicines. In total, 2200–2300 every month.* (60 years, male patient)

*MDR-TB patients face difficulty in finding someone to give them injections. Even if there are doctors, they charge a minimum of 20, 30, or 50 rupees for giving one injection… It might also happen that patient has to travel far off to get those injections.* (TB health visitor, 5 years of experience)

##### Patient factors

The patient's denial of TB, the perceived unavailability of services, and having the first consultation with a private doctor were perceived as the reasons for increased costs due to TB. Choice of combination medicines, frequent sickness, and lowered immunity was the patient factors responsible for higher costs due to care of diabetes mellitus. Private care-seeking was specifically observed for the care of diabetes among patients with TB-diabetes comorbidity.

*Patients say that ‘Sir, you suspected directly TB only? None of my family members have TB. None in our family had TB in seven generations.’* (District TB officer, 20 years of experience)

*Costs go up because they have first taken treatment in private, all investigations must have been done from top to bottom. Same way, when they will come back into govt., then everything will be repeated. Double costs, long duration of treatment, maybe treatment failure, and maybe loss to follow up—chances of all these will increase.* (District program coordinator, 1.5 years of experience)

*For diabetes, drugs are available in combination in private, whereas in govt. they are available as two separate drugs, so patients have to take 2 pills. Due to this, some patients say that they are taking a combination drug from private for many years and are used to it, so they don’t want to take medicines from the government.* (Senior treatment supervisor, 18 years of experience)

##### Cash assistance problems

Insufficient/ no cash assistance, no reimbursement of transport fare, and lack of documents were the problems perceived regarding availing of cash assistance. It is not only the lack of basic documents like an *Aadhaar card* (social security number) or bank account number but also the requirement of caste and income certificates for availing of the benefits of the social welfare scheme, that acts as an impediment. Patients ultimately end up incurring travel costs for procuring and submitting these documents.

*Since I am a driver in a school, I am managing. The rest of the retirees do not get help. I do not know how long it has been since the 500–500* [Indian rupees] *help was given to us. I have given the bank account detail twice there but still, the money was not received. Years passed but still not received.* (60 years, male patient)

*My father had to sell all jewelry, it was his life’s earnings. He had borrowed the money on interest and made a hand cart for selling seasonal foods to meet ends. I received 500 rupees per month for milk and food. We spent 650 rupees to get milk, we had to spend extra 150/- rupees every month.* (24 years, female patient)

*I fill out forms at the health center to provide the benefit of 500 rupees to patients. But, they compulsorily ask for patients' caste certificates and income certificates. Some patients don’t have it and others have expired certificates. Such patients have to incur travel costs to renew the certificates.* (TB health visitor, 2 years of experience)

##### Job loss

Patients, especially during the initial stages of the disease, are unable to go to work and thereby incur losses. COVID-19-related lockdown increased the losses of patients due to the interruption of work.

*I could not stand during the start of TB treatment for 2–3 months as my body became very weak. I have not worked for these many months.* (69 years, male patient)

*I have to pay borrowed money by doing work at others’ homes. Due to the lockdown, there was no work for my husband and son for three months, both stayed at home during this period. We worked at others’ houses to earn our bread and butter.* (45 years, female patient)

##### Cost of monthly visits

For the care of diabetes, patients have to make frequent and monthly visits to health facilities. Patients incur costs for travel, consultation, and special investigations during such monthly visits. Some of these costs were incurred for the care of TB as well.

*Every month I have to visit for medicines and report, which costs 1700* [Indian rupees] *per month. For diabetes, I consult a private doctor, as I don’t know that the medicines and reports are available at government* [health facilities]*.* (60 years, male patient)

*For diabetes, the patient’s routine check-up is being done. So, even if they incur costs, they have to bear them. There is no other option… they need to come for a test and medicines. Even if we send our staff to their home, we can only provide RBS* [random blood sugar] *or at the most their medicines. But, they have to remain in consultation* [with a doctor] *monthly.* (Senior treatment supervisor, 20 years of experience)

#### Solutions for increased costs of TB-diabetes

The program functionaries perceived preventing delays in care, appropriate guidance, and home-delivered care (for diabetes) as some of the solutions for reducing the costs (Table 4). Health system strengthening, cash assistance, and timely referral were the solutions perceived by the patients (refer to Supplementary Table S6 for a description of each code).

**Table 4:** Framework analysis describing categories of solutions for increased costs incurred due to TB and diabetes in Bhavnagar.

Categories of solutions for increased costs	Program functionaries’ perception		Patients’ perception	
	TB	Diabetes	TB	Diabetes
Prevent delays in care	√			
Private sector improvements	√			
Appropriate guidance	√	√		
Cash and other assistance	√	√	√	√
Involve frontline workers	√			
Health system strengthening	√	√	√	√
Home-delivered care		√		
Timely referral			√	

##### Prevent delays in care

The program functionaries perceived that the delays in the care provided to the patients can be avoided through early diagnosis, quicker dissemination of reports of investigations, obtaining spot sputum samples from the patients, and strengthening the bi-directional screening for TB-diabetes.

*All services are available in our government set up, but not on priority. If sonography is to be done, their turn comes after four days. Now, if the patient is suspected to have abdominal TB, then the sonography should be done on the same day itself. Only if early diagnosis is done, it will help control TB.* (Senior treatment supervisor, 20 years of experience)

*Especially for extra-pulmonary TB and sputum-negative TB, all the services need to be provided on a priority basis. Otherwise, they are going to incur costs in private. For MRI* [magnetic resonance imaging]*, two options are being given—one at our hospital* [for delayed reports] *and the other at a private hospital for quick reports.* (Senior treatment supervisor, 20 years of experience)

*If we include 100% patients with diabetes for the screening of TB, then we can diagnose a good proportion of patients suffering from TB. But, it is not happening as of now and is needed.* (Senior treatment supervisor, 20 years of experience)

##### Private sector improvements

The costs incurred by patients can be significantly reduced if private practitioners guide the patients on the availability of free care and cash assistance in the public sector. In addition, private doctors should refer and notify patients whom they foresee at high risk of defaulting on their treatment or, would not be able to afford the costly treatment.

*Some general practitioners are good, they give the option of taking AKT* [anti-TB treatment] *either from the government or from them. For example, there is one private doctor… who notifies his patients regularly but, doesn’t give the address of any patient. These patients do not come to know that the treatment is free in government or, that monthly 500 rupees assistance is being given.* (TB health visitor, 6 years of experience)

*Private doctors inform us and also assess the condition whether the patients are capable to pay and whether they will be able to complete the treatment course or not. If they feel, immediately they refer the patient to DTC* [district TB center] *or to government hospital explaining that the treatment is free over there.* (Senior treatment supervisor, 5 years of experience)

##### Appropriate guidance

Counseling patients on the importance of completing treatment and creating faith in the quality of medicines would help reduce the costs incurred by the patients. Also, patients with TB-diabetes need to be guided on the availability of free care in government health facilities. For patients with TB, all assistance and care available for a patient registered in the public sector can also be availed for free by patients registered under a private practitioner.

*Frontline health workers conduct daily house-to-house visits. If the health workers provide the correct guidance to a suspect TB patient—that for these symptoms, this is the place where the patient would get correct treatment—if they are not told this, then they will run to private doctors or will not go at all or will default on treatment.* (District TB officer, 20 years of experience)

*Community participation is very important for this program. People themselves need to understand that these medicines are available free of cost and are of good quality and on completing this treatment, TB gets completely cured. Until this community participation is not there, they will incur costs.* (Senior treatment supervisor, 5 years of experience)

*They are taking the treatment of diabetes from private, then they are detected with TB, so now they came in our system. They might be on Metformin, Glipizide, or any other drug. To reduce the costs which they are incurring, we suggest they take those medicines from us.* (Senior treatment supervisor, 8 years of experience)

##### Cash and other assistance

Patients, as well as program functionaries, reiterated the need to continue the cash transfer program and social welfare scheme as it had helped the patients significantly. There were also demands to timely disburse the cash assistance, increase the existing cash assistance, provision of nutritious food kits, and reimbursement of costs, mainly transport fares.

*It often happens that the bank account is not valid. Either a cash system should be provided or a card should be provided through which they don't have to pay anywhere. Even in government bus, if he is getting free transport, then with that greed also, they will come for consultation.* (District program coordinator, 1.5 years of experience)

*We have benefited a lot from the cash assistance… I was able to purchase fruits, milk or any other food item as per the requirement of the medicines.* (66 years, male patient)

*If patients do not have sufficient food to eat even once at their home, then there should be some increase in the 500 rupees assistance or, reimbursement of some costs incurred by them. Because, if food also goes into the stomach, then medicines will work better. Many times, we have coordination with some trust organization who give us a kit comprising of cereals, pulses, jaggery, oil—which is important for the patient during the illness.* (TB health visitor, 5 years of experience)

##### Involve frontline workers

The frontline workers of public health institutions have to be regularly motivated to consider the TB program as one of the many other programs for which they are currently working. They should also be provided with work-based incentives from the TB program. The support of staff working under the TB program was well applauded by the patients, however, the program functionaries felt that certain mechanisms for administering injectable anti-TB drugs at the home of patients were needed.

*Some patients, residing in remote locations, have to go to distant places to get certain injectable drugs such as Kanamycin. Just as we have treatment supporters for delivering medicines, if we have some qualified person to give them injections, then their cost of transportation can be saved.* (District program coordinator, 1.5 years of experience)

*As of now, the only incentive is for their role as a treatment supporter. Otherwise, for field visits or active case finding, they do not get any incentives.* (Senior treatment supervisor, 20 years of experience)

##### Health system strengthening

Program functionaries suggested increasing facilities for sputum microscopy, strengthening sub-health centers, reducing delays, and improving care from providers. Patients with diabetes purchase medicines from private chemists as the fixed-dose combination are not available in the government setup. Program functionaries thought that these combination medicines should be made available for free in the government setup.

*If we can strengthen the grass-root level sub-centers, if we can make all the services available through ASHA* [trained female community health activist]*, then whatever traveling of 2–5 km they have to do currently can be avoided.* (District TB officer, 20 years of experience)

*After the opening of a new urban health center in our area, medicines and laboratory investigations are available here. I can get tested here and can consult a doctor too. It is just 1.5–2 km away. The rickshaw fare is only 10 rupees… We only have to visit it one time and the time taken is also less.* (36 years, female patient)

*For diabetes, if the combination of medicines is available in a single tablet from government, then the patient can take treatment from the government and that way their costs can be reduced.* (Senior treatment supervisor, 7 years of experience)

##### Home-delivered care

As is being done under the TB program, program functionaries perceived that medicines for diabetes can also be home-delivered through village-level frontline health workers.

*If a patient is taking regular diabetes medicines, then we can reach their home and give their treatment… If we want, this kind of arrangement is possible to do because, in every village MPHW* [multipurpose health worker], *FHW* [female health worker], and *ASHA* [accredited social health activist] *are available.* (Senior treatment supervisor, 7 years of experience)

##### Timely referral

Patients perceived that if private doctors refer them early to government hospitals, then they would not incur more expenses.

*We were taking medicines from a private doctor. But, later, he informed us that the medicines are all the same, so you take them from the government. Then we went to the government, so, no expenses were incurred.* (66 years, male patient)

## Discussion

The costs incurred due to TB have the potential to push families below the poverty line^14^. Moreover, TB mostly affects individuals with low incomes and the working population^13^. Thus, when the sole earner of a low-income household has TB, it is a catastrophe. The vicious cycle of poverty, malnutrition, and TB infection continues^14^. Because we believed that individuals with diabetes likely seek care in the private sector, we hypothesized that diabetes care might significantly contribute to the TB catastrophe. To summarize our study findings, patients with TB-diabetes incurred lower costs in our study setting as compared to their income. Costs due to diabetes marginally increased the chances of catastrophic costs due to TB. Even with lower costs, a few patients employed negative financial coping strategies such as borrowing money to cover anticipated costs. Health system strengthening, an increase in cash assistance, and other benefits such as a nutritious food kit were suggested both by program managers as well as the patients for reducing the costs incurred due to TB-diabetes.

In our research, the median (IQR) cost spent by patients with TB was INR 618 (378–1933) [US$ 9 (6–28)]. This was less than the INR 10,000 [US$ 147] median cost indicated in a recent nationwide study conducted in India^22^. In our study setting, we also observed that 4% (95% CI 3–7%) of patients experienced catastrophic costs attributable to TB. This figure was comparable to a few studies in India^8,23^, but lower than most other published research in metropolitan areas, which found prevalence rates of up to 68%^9,24^. Our study setting mainly consisted of semi-urban and rural settings. The care for TB is decentralized through a network of public health institutions (PHIs) in India. The medicines are placed nearby their homes under the directly observed therapy (DOT) supervision of treatment supporters. Four out of five patients in our study first visited a government health facility for their TB care, most of the diagnostics and treatment is free in the public sector. Almost all patients had less severe (drug-sensitive, pulmonary) forms of TB. Although patients from rural settings might have incurred higher transport costs, patients in urban areas must have incurred lower costs due to the proximity to health centers. These reasons explain the lower costs incurred due to TB in our study.

The median (IQR) cost of combined TB-diabetes care in our research setting was INR 1314 (788–3170) [US$ 19 (12–47)]. There was no comparable research on combined TB-diabetes expenses from India to corroborate our findings. However, researchers from outside India documented a higher proportion of catastrophic costs due to concurrent TB-diabetes comorbidity^10,11^. In our study setting, patients who were first diagnosed with diabetes and later TB chose to seek care in the private sector. When such people develop TB, they realize that the government also provides diabetes medications. They do not, however, go to the government sector because they are already compliant with the private treatment regimen for diabetes. Patients with diabetes are conventionally put on a two-drug regimen, the combination of which is available as a single tablet in the private sector but not at government health centers. Patients prefer consuming a single tablet for diabetes and avoid shifting to government drugs as they are accustomed to them. Moreover, as patients with diabetes are required to follow up with their physicians on monthly basis, costs (transport, laboratory, medicines, and others) must have been incurred for these visits. These incremental costs of diabetes might explain the marginal rise in the prevalence of catastrophic costs due to TB among comorbid patients in our study.

Even when the government provides free TB and diabetes care, as in our study, the Kyrgyz researchers reported co-payments^10^. They further reported lower catastrophic spending for TB-diabetes comorbid patients (8%) than for TB-only patients (19%), which was discrepant with our study^10^. This disparity might be explained by the fact that they estimated catastrophic spending/expenditures using different cut-offs than our study^10,11^. In addition, patients with TB in Kyrgyzstan are treated in-patient for two to three months, followed by out-patient therapy for four months, as opposed to patients in India, who are primarily treated at home^11^. Their second study found higher odds of catastrophic health spending among patients with TB-diabetes comorbidity as compared to patients with TB^11^, similar to the present study. When compared to individuals with either disease, TB-diabetes comorbid patients visit the hospitals the most for follow-up^10^. Similar to our study, patients with diabetes in Kyrgyzstan seek care from private practitioners, which requires patients with TB-diabetes comorbidity to attend two separate facilities—private for diabetes care and government for TB care^11^. Thus, as observed for patients in Kyrgyzstan, the costs of travel and wage loss experienced during these hospital visits had an additive impact on TB-diabetes co-affected individuals in our study setting^11^. Finally, several patients with TB-diabetes comorbidity in our research borrowed money or began working to meet the costs. These coping mechanisms, which serve as a proxy for catastrophic costs incurred, imply that reliable sources of money, such as income, are used to fund the cost of care and are frequently supplemented with additional financial assistance, highlighting the need for a tailored cash assistance program for comorbid TB patients^25–27^.

The analysis of the in-depth interviews pointed toward the requirement of concerted and coordinated efforts of multiple stakeholders for bringing about a reduction in the costs incurred. Patients generally complain about long queues and delays in receipt of appropriate care at government health facilities, especially at tertiary care hospitals. To avoid delays in receiving care and to save the loss of their work time, patients seek care at private clinics. However, they end up incurring higher costs (for investigations, drugs, consultation, and others) at private clinics than the anticipated savings by avoiding wage loss. Thus, delays in caregiving need to be minimized at government facilities and awareness needs to be generated on the option of availing all the benefits for free to patients under private consultation. Private practitioners, on the other hand, should timely refer patients whom they perceive as unable to afford treatment for a long duration. Program functionaries, as well as patients, also suggested increasing the cash assistance for patients with TB-diabetes comorbidity for the timely purchase of nutritious food. They also suggested providing a nutritious food kit that would help them to tolerate the adverse drug reactions and help in completing their treatment^28^. A universal cash transfer scheme would help alleviate poverty, secure access to food, and increase the corpus available to patients for spending on healthcare^29–31^.

Collaborative activities between TB and comorbidities have the potential to ensure optimal resource utilization, minimize healthcare visits, improve treatment outcomes, and hence lower patient costs^32,33^. In 2017, India launched the joint framework for TB-diabetes activities, however, the bi-directional screening and management for TB-diabetes under programmatic settings needed improvement^5,34^. Apart from generating awareness of the relationship between TB and diabetes, each TB patient should be tested/managed for diabetes, and each diabetic patient should be tested/managed for TB^5,35^. According to the program functionaries interviewed in our study, the former was adequately executed, whereas the latter needed improvement. Strengthening the joint TB-diabetes activities will ensure early diagnosis and prompt treatment of either disease, while providing quality care will ensure successful management of such individuals, decreasing costs^10^.

### Strengths and limitations

This study adds to the currently scarce body of evidence on costs incurred by patients with TB-diabetes. A major limitation of the research is the absence of a comparison group of TB patients without diabetes. Patients were enrolled in the study as early as four years before data collection, making it difficult for them to remember any costs they may have spent; as a result, we may have underestimated the costs incurred. People in India mostly work in informal sectors, and our study relied on self-reported annual household incomes, which are thought to be lower than estimates based on assets, consumption, or expenditure, potentially leading to a biased estimation of costs. Although diabetes is a chronic disease, its costs were assessed only for the duration of TB treatment. Also, it is possibile that not all patients were tested for diabetes, or that a complete history of current diabetes was not gathered as part of the TB program, resulting in a non-representative sample.

Nearly 40 patients were started on TB treatment during the first wave of COVID-19, which would have raised their transportation costs and visits to private facilities, increasing the likelihood of incurring higher costs. The study’s omission of individuals with TB who were registered in the private sector may have led to an underestimation of the costs incurred. In our qualitative component, we did not interview healthcare providers, who are key stakeholders and would have offered unbiased inputs. Nevertheless, we feel that interviewing program staff and patients would have resulted in an accurate triangulation of responses. We adhered to the guidelines for reporting observational studies and qualitative research^36,37^. The findings of our study can be generalized to similar semi-urban and rural settings in India.

## Conclusions

We conclude that patients incurred lower catastrophic costs due to TB in our study setting. However, in addition to a marginal increase in the percentage of catastrophic costs, co-existent diabetes nearly doubled the median total costs incurred among patients with TB. Strengthening the collaborative TB-diabetes activities, tailoring the cash transfer scheme for comorbid patients, and making the common two-drug combination diabetes tablets available at government drug stores would help TB-diabetes comorbid patients cope with the costs of care. Future research should compare the effectiveness of cash assistance schemes vs. universal cash transfers in lowering catastrophic costs, as well as determine whether collaborative TB-diabetes activities reduce catastrophic costs.

## Supplementary Information

Supplementary Information.

## Data Availability

The datasets generated and/or analyzed during the current study are available upon a reasonable request to the corresponding author.

## Abbreviations

AKT: Anti-Koch’s treatment
DOT: Directly observed treatment
COVID-19: Coronavirus Disease-2019
INR: Indian rupees
IQR: Interquartile range
NPCDCS: National Program for prevention and control of Cardiovascular diseases, Diabetes, Cancer, and Stroke
NTEP: National Tuberculosis Elimination Program
PHI: Public Health Institution
SLI: Standard of living index
TB: Tuberculosis
