# Supplementary material for: A sequential explanatory mixed-methods study on costs incurred by patients with tuberculosis comorbid with diabetes in Bhavnagar, western India
Source: Sci Rep. 2023 Jan 4;13:150. doi: 10.1038/s41598-023-27494-7 (PMC9811877; doi:10.1038/s41598-023-27494-7)
Supplement: Supplementary file 1 — Supplementary Information. [file 41598_2023_27494_MOESM1_ESM.pdf]

Title: A sequential explanatory mixed-methods study on costs incurred by patients with tuberculosis comorbid with diabetes in Bhavnagar, western India

Supplementary files

Supplementary Table S1: Percentage and median (IQR) direct medical costs in Indian Rupees (INR) incurred by patients with >0 direct medical costs from January 2017 to December 2020 in Bhavnagar

Groups	n (%)	Median (IQR)
**TB**	58 (19)	1800 (700-6888) [US$ 26 (10-101)]
**Diabetes**	30 (10)	1050 (295-3725) [US$ 15 (4-55)]
**TB-diabetes**	64 (21)	2300 (753-8863) [US$ 34 (11-130)]

Supplementary Table S2: Association between >0 direct medical costs and first consultation visit at a private facility from January 2017 to December 2020 in Bhavnagar (n=304)

Groups	Cost groups	First consultation at private facility n (%)	First consultation at government facility n (%)	Total	Chi-square test	p-value	Odds ratio (95% CI)
**TB**	**>0**	55 (95)	3 (5)	58 (100)	272	<0.001	2237 (365-13707)
	**≤0**	2 (1)	244 (99)	246 (100)			
**Diabetes**	**>0**	25 (83)	5 (17)	30 (100)	174	<0.001	147 (49-473)
	**≤0**	9 (3)	265 (97)	274 (100)			
**TB-diabetes**	**>0**	61 (95)	3 (5)	64 (100)	258	<0.001	956 (222-4110)
	**≤0**	5 (2)	235 (98)	240 (100)			

Supplementary Table S3: Catastrophic costs incurred by patients with TB comorbid with diabetes during January 2017 to December 2020 in Bhavnagar (n=304)

Cut-off percentage of annual household income	Percentage (95% *CI*) of households facing catastrophic costs due to TB	Percentage (95% *CI*) of households facing catastrophic costs due to TB-DM
20	4 (3-7)	5 (3-8)
15	5 (3-8)	8 (5-11)
10	7 (4-10)	11 (8-15)
5	13 (10-21)	18 (14-22)

Supplementary Table S4: Coping strategies employed by patients with TB comorbid with diabetes during January 2017 to December 2020 in Bhavnagar (n=304)

Type of impact	Number (%) or median (IQR)
Coping strategy of any kind	16 (5)
Borrowed money as loan	6 (2)
Amount borrowed in Indian Rupees	10000 (5000-12500)
Lost employment after TB diagnosis	8 (3)
Started employment to cover costs of TB	2 (1)
Working days lost to TB	45 (30-60)

Supplementary Table S5: Description of codes for reasons of increased costs incurred by patients with TB comorbid with diabetes in Bhavnagar

**Reasons for increased costs of TB-DM**		
**Categories**	**Code**	**Description of code**
Costs in govt. hospitals	Consultation time	Patients have to spend 1-2 days' time when they visit the tertiary care hospitals for consultation. For TB-DM comorbid patients, this time increases to 2-3 days. This loss of time affects their occupation and leads to loss of wages.
	Inaccessible for rural patients	Patients in rural areas do not have access to tertiary-level healthcare facilities. They have to travel to cities for the same. They have to arrange for a special mode of transportation or, have to incur higher transportation costs for reaching the hospitals.
	Delayed diagnosis	Patients generally make a few visits to other health facilities (including private) before they are diagnosed at the tertiary care hospital. This delays

		their diagnosis and thereby the initiation of treatment.
	Inadequate manpower	There is paucity of specialists in rural areas, forcing patients to travel to tertiary care hospitals. Also, the staff appointed for home visits of patients with TB (health visitors and senior treatment supervisors) have to cover a large geographical area, leading to their inability to visit all patients.
	Lack of frontline worker involvement	Due to the shortage of TB staff at the ground level, frontline health workers have an important role to play in supplementing the TB program during their routine home visits. However, program functionaries of TB perceived that the frontline health workers, despite being pushed, were not doing enough for the TB program mainly due to overburden of other national health programs.
	Lack of govt. health centers nearby	Not only rural patients, but, patients in urban areas also perceived that lack of govt. health centers nearby forced them to consult private doctors nearby to their home.
	Delay in tertiary care hospitals	Patients avoid visiting tertiary care hospitals due to the long queues. To save time and to avoid work loss, patients visit private doctors instead.
	Transport	Transport fare for rickshaw or bus for clinic visits was the one of the commonest cost incurred. Patients with diabetes had to incur higher costs due to the requirement of monthly visits for collection of medicines or for follow-up.
	Frequent visits	For diabetes, patients have to visit the health facilities every month. Generally, these are medicine collection visits.
	Lack of afternoon outpatient department (OPD) in govt.	Patients visit private facilities due to the lack of afternoon OPD in govt. hospitals.
Private sector costs	Greed of private practitioners	Patients felt that the private practitioners continue to treat them till they are able to pay, instead of referring them timely to the govt. health facilities. The greed of private practitioners to leech the patients till the last penny on them was perceived as one of the important reasons for increase in costs of patients.
	Local healers	For most of the diseases, patients want a quick cure, within 24-48 hours of starting treatment. For TB too, patients seek to get better at the earliest. Because of this psychology, patients tend to seek local healers and sometimes also stop their medicines.

	Laboratory investigations	Patients have to incur costs for laboratory investigations, especially when they seek care at private health facilities. Sometimes, when the facilities are not available in govt., patients are referred to private health facilities for investigations.
	Medicines	TB patients seeking care from private facilities incur costs for drugs. The program functionaries perceived that patients get medicines for TB from govt., but prefer to get anti-diabetic drugs from private. This is mainly true for patients who are already taking treatment for diabetes from private doctors, and are subsequently diagnosed with TB in govt.
	Cost of injections	Patients, especially those with multi-drug resistance TB, have to pay for getting injections administered from either a doctor or a nurse.
	Consultation fees	Patients seeking care at private sector have to pay the consultation fees of doctors.
	Less referrals	Despite govt. circulars, the referrals from private practitioners to the govt. are very less.
	Late detection	Late detection of disease, with prescription of multiple investigations, was observed among private practitioners by program functionaries.
	Private practitioners	Private practitioners charge patients for consultation, laboratory investigations, medicines, and others. Once patients are not able to manage financially, then the private practitioners refer them to govt. health facilities.
	Radiology	Patients have to pay for X-ray in case of pulmonary TB. In case of extra-pulmonary TB too, expenses are incurred for UGS, CT-scan, and other radiological investigations.
	Delayed diagnosis	Patients perceived that private practitioners delayed the diagnosis for making profits from the payments made by the patients for consultation fees, and others.
	Trial AKT	Private practitioners prescribe anti-tuberculosis treatment for shorter than recommended duration, on trial basis. Even for shorter duration of prescribed treatment, patients have to sell their jewellery to cope with the costs of private medicines.
Patient factors	Patient denial of TB	Patients deny that they have TB. They believe that since their immunity is good they can’t have TB. Due to this, patients keep taking antibiotics from a private GP. They get diagnosed 2-4 months late.

	Perceived unavailability of services	Patients, especially in villages, believe that govt. health facilities are closed during evening/ night time. Therefore, they visit a private clinic.
	More faith in private sector	Patients have a certain kind of resistance towards government. Despite the facilities being offered by govt., patients first try taking medicines from private. They believe that the medicines provided are better in private.
	First visit at private	The costs incurred by patients increase because they first visit a private practitioner. When the patients go back to govt. setup, all the investigations would be repeated. Patients would have to spend double costs, longer treatment duration, and may also end up with treatment failure or may also stop treatment.
	Quick cure	Patients want speedy results. Their psychology is such that they want to get symptomatically well within 2 days, and cured within 5 days. Once the recovery is delayed, they either stop treatment or search for an alternative cure.
	Lowered immunity	Patients with diabetes complained of low immunity and inability to work due to the illness. This led to wage loss and increase in costs.
	Combination medicines (Diabetes Mellitus)	For diabetes, patients are generally more comfortable with consuming combination medicines, which they purchase from private. They continue their consultation too from a private doctor for diabetes. Patients prefer consuming a single tablet, instead of 2 separate anti-diabetic drugs given in govt.
	Frequent sickness	Patients with diabetes got sick very frequently. Frequent sickness increased their hospital visits, and thereby their costs.
	Change of doctors	Patients shift to private sector in case of non-recovery. Once the costs incurred in private sector becomes unaffordable, patients come back to the government sector. Ultimately, patients end up spending more money during their care.
	Confidentiality	Patients with comorbidity of TB and diabetes, seek care of diabetes from private practitioners to keep their status of TB confidential.
	Extra-pulmonary	Extra-pulmonary TB, due to its nature of disease, usually gets diagnosed using advance techniques in the private sector. Such patients are then referred to the government setup for further management. By the time patients reach the government health facility, they incur costs in the private sector for their care for extra-pulmonary TB.

	Private care seeking	Patients seek care from private practitioners for TB as well as for diabetes. Among comorbid TB-diabetes patients, patients preferred to seek care for diabetes from private doctors and for TB from government setup. However, for TB, the care seeking behavior towards private during the initial part of the diagnosis led them to incur costs.
	Nutritious food	Patients incurred costs due to the necessity of consuming nutritious food such as milk during the course of treatment of TB. Due to loss of appetite while being on an anti-TB drug regimen, patients preferred to consume milk/ fruits to maintain immunity status.
	Lack of awareness	For TB, patients have a general lack of awareness about the possibility of the disease even with mild symptoms such as cough. For diabetes, patients were not aware about the availability of free medicines in government health facilities.
	Adverse drug reactions	Patients complained about lack of respite from side effect of TB medicines despite multiple consultations, both in private as well as in government. Patients incurred costs for purchasing additional medicines, sometimes Ayurvedic concoctions, for getting relief from the adverse drug reactions.
	Delayed care seeking	Due to the inability to recognize symptoms such as cough as a possible indication for TB, patients sought care only when the disease progressed to advance stages or only when the symptoms aggravated. Similarly, some patients sought care for diabetes only when the problem (of high blood sugar) aggravated.
Cash assistance problems	Insufficient cash assistance	Patients spend over and above the 500 Indian rupees which they receive monthly under the cash transfer program. Patients perceive the assistance to be insufficient to even purchase milk for the entire month.
	Lack of documents	Patients are not able to avail for the cash assistance due to lack of bank account number or lack of *Aadhaar card* (social security number). Also, patients have to go from post to pillar for procuring certain documents (like caste and income certificate) required for availing the benefit of social welfare scheme. Patients incur travel costs while procuring such certificates from government departments.
	No transport reimbursement	Patients with drug-susceptible TB do not receive reimbursements for the transport fare paid by them during clinic visits.

	No cash assistance	Some patients complained of non-receipt of cash assistance even after submission of the requisite documents.
Job loss	Wage loss	Patients, especially during the initial stages of the disease, are unable to go to work and thereby incur losses. Due to weakness and sometimes due to adverse drug reactions during the initial 2-3 months of anti-TB treatment, patients are forced to rest at home.
	COVID-related work loss	COVID-19 pandemic wreaked havoc among everyone's lives. The lockdown imposed during the pandemic increased the losses of patients as employment stopped for almost everyone. Patients complained of their inability to pay off debts during this period.
Costs of monthly visits	Transport	Patients pay for the transport fare while visiting the health facilities. These costs are incurred both for the care of TB as well as diabetes. The transport fare is higher for patients residing in rural areas as they have to come to city areas where the district hospitals are situated.
	Frequent visits	Although patients with TB and diabetes avail services from nearby health centers, they have to visit tertiary care centers for the follow-up visits.
	Monthly visits	Program functionaries perceived that patients with diabetes have to be monitored monthly for their control of blood sugar levels. Although services such as random blood sugar test and medicines for diabetes can be delivered at home, it was necessary to have regular consultation with the doctors.
	Consultation time	Patients have to stand in queue for investigations, for consultation, for medicines, and others, at the tertiary care hospitals. Program functionaries perceived that this consumes around 2-3 days of the patients' time, especially during the initial phase of diagnosis and stabilizing on treatment.
	Special investigations	For certain investigations which are not available at primary health centers, the patients have to visit the tertiary care health facilities. Many a times such tertiary care hospitals are located in urban areas. Thereby, patients incur costs for transport and may have to lose their daily wages.

Supplementary Table S6: Description of codes for solutions for increased costs incurred by patients with TB comorbid with diabetes in Bhavnagar

**Solutions for increased costs of TB-DM**		
**Categories**	**Code**	**Description of code**
Prevent delays in care	Early diagnosis	Early diagnosis would help in early initiation of treatment and thereby reduction in costs incurred by the patients. Early diagnosis would also help in control of TB.
	Quick reports	Program functionaries perceived that results of any investigations should be given as quickly as possible to the patients. They perceived that we can save patient’s time and they need not wait for hours together for their reports.
	Spot samples	It cannot be predicted whether the patients would come back for testing of another sputum sample. Thereby, program functionaries opined to take spot sputum samples from the patients wherever the patient might be and whenever possible.
	Bi-directional strengthening	Program functionaries perceived that all patients with TB are being tested for diabetes. However, all patients with diabetes are not being tested for TB, which is needed. Suitable mechanisms need to be explored for strengthening the bi-directional screening for TB-diabetes.
Private sector improvements	Correct guidance	Program functionaries perceived that the costs of patients can be reduced if private doctors provide correct guidance to the patients. Private practitioners should guide the patients regarding free treatment and other benefits like cash assistance available in the government setup.
	Referral	Some private doctors refer patients whom they foresee as high risk for defaulting on their treatment or, would not be able to afford the costly treatment. Program functionaries show the notification of such patients under the private doctor itself, however, provide treatment as well as other benefits as a patient of public sector.
	Increase notifications	By increasing notifications from the private sector, program functionaries would be able to provide benefits such as cash assistance to patients being treated in the private sector. The private doctors are also benefitted through notifications as they are disbursed 500 Indian rupees for every patient that they notify.
Appropriate guidance	Awareness generation	Program functionaries perceived that awareness generation activities need to be multi-pronged. Awareness regarding sign/ symptoms of TB,

		completing full course of treatment, consulting health workers in case of adverse drug reactions, and others, would help patients reduce the costs incurred by them.
	Guidance on free treatment in government	Program staff of TB as well as other health workers should guide patients regarding free treatment in government setup. Patients with either TB or diabetes or both should be counselled on the availability of free care in govt. health facilities.
	Guidance on cash assistance for private patients	Patients need to be explained that even if they are taking treatment for TB from a private doctor, they are eligible for cash assistance of 500 Indian rupees every month till the completion of treatment.
	Guidance on services for private patients	Patients also need to be counselled on the services available for patients with TB taking treatment from a private doctor such as testing for HIV, testing for diabetes, regular home visits by TB program staff, and others.
Cash and other assistance	Transport reimbursement	Reimbursement of transport fare of patient and one accompanying member is being provided for MDR-TB patients, but not for drug-sensitive TB patients. Program functionaries suggested the government to provide reimbursement of transport fare to all TB patients, the expenditure being entered into a card or by providing a ‘free travel’ card.
	Cash transfer program	Patients used the cash assistance of 500 Indian rupees per month till treatment completion on purchase of milk, fruit and other foods which helped them tolerate the adverse drug reactions of anti-TB medicines. Also, some patients felt that the cash assistance acted as a cushion during the time they were not able to go to work.
	Social welfare scheme	In addition to the cash assistance of 500 Indian rupees under the Nikshay Poshan Yojana, patients with TB belonging to scheduled tribe (ST), scheduled caste (SC) and other backward classes (OBC) are eligible for additional 500 Indian rupees per month under the social welfare scheme for a period of 6 months. Program functionaries perceived this scheme as beneficial for the patients and the need to continue it.
	Nutritious food kit	Nutritious food kit was perceived to be an important adjunct to the medicines by both patients as well as program functionaries. Program functionaries highlighted the importance of nutritious food kit in ameliorating the side

		effects of anti-TB medicines and for improving immunity.
	Timely cash assistance	Timely cash assistance would help patients to spend it on purchase of food or clearing off their debts.
	Increase in cash assistance	It was suggested to increase the cash assistance as the existing amount was perceived to be less to purchase food throughout the course of anti-TB treatment.
	Assistance for buying nutritious food	It was also suggested to provide assistance for buying nutritious food. Program functionaries perceived that the existing cash assistance of 500 Indian rupees per month got expended on paying the transport fare or overcoming the wage loss during clinic visits. Thus, additional assistance for purchasing nutritious food was required.
	Reimbursement of costs	Different types of costs incurred by the patients, such as transport, wage loss, investigations, and others, should be entered in a chart and should be reimbursed through well thought mechanisms like patient welfare committee of a government hospital.
	Cash assistance	Patients with diabetes demanded cash assistance to overcome their costs incurred for monthly investigations and medicines. They perceived that in presence of additional diseases such as diabetes among patients with TB, additional cash assistance should be provided to afford food and to pay off their debts.
Involve frontline workers	Staff motivation	Field workers working in TB program and other frontline workers need to be motivated to give their level best in their work. Frontline workers of public health institutions, generally have an impression that TB program has its own dedicated staff. They tend not to include any part of the TB program in their routine visits. The frontline workers of public health institutions have to be regularly motivated to consider TB program as one of the many other programs for which they are currently working.
	Frontline worker incentives	The frontline workers of public health institutions should be provided with work-based incentives from the TB program. Currently, they only get incentives for their role as a treatment supporter. In addition they get incentives if a sputum sample of a suspect patient, sent by them, turns out to be positive. But, if the sputum samples test negative, they don’t get any incentive.
	Injections at door-step	Program functionaries felt that injectable anti-TB drugs such as Kanamycin should be administered

		at the homes of the patients. Patients, especially in rural areas, have to travel long distances just for getting such injections.
	Health worker support	The support provided by the health worker staff of TB program was applauded by the patients. Patients perceived that the health workers used to regularly take follow-up visits and counselled them as and when needed.
Health system strengthening	Increase lab facilities	Although the number of laboratories have been increased, program functionaries felt that there was a need to increase facilities of sputum microscopy in the existing laboratories.
	Strengthen rural centers	Program functionaries suggested to strengthen village-level health facilities such as sub-health centers and to deliver all services through ASHA (trained female community health activist) volunteers in the village.
	Improved facilities nearby	For patients residing in rural areas, all the diagnostic as well as treatment facilities should be made available nearby their villages so that their costs can be reduced. Even in urban areas, patients prefer to visit the nearby health centers as it takes less time as compared to the time taken at tertiary care hospitals.
	Improved care	Empathetic care should be provided to the patients. Patients preferred to consult the same doctor from beginning of the treatment till the end. Patients also asked doctors to pay a little more attention and provide better care.
	Increase facilities	Patients wanted all facilities available in private to be made available in government hospitals for free. Without any hassles, patient should get medicines from time to time, and investigations should be made free of cost.
	Public healthcare seeking	To save time and avoid loss of work, patients tend to consult a private doctor first. However, if patients go to government hospital on the first visit itself, then they perceive that they would not incur much costs.
	Reduce delay	Patients want government health facilities to reduce the delay in giving reports of investigations. Patients are always met with a long queue at government hospitals. They lose their work on each clinic visit.
	Decentralized care	For TB as well as for diabetes, most of the care can be availed at a nearby health centre, however, for certain investigations/ follow-up, patients have to visit a tertiary care hospital. If all diagnostics and medicines are made available at a

		nearby health center, then patients need not visit a tertiary care hospital.
	Free combination medicines (Diabetes Mellitus)	Patients with diabetes purchase medicines from private chemists as the fixed dose combination is not available in government setup. Program functionaries were of the opinion that these combination medicines should be made available for free in the government health centers.
	Easy availability of medicines (Diabetes Mellitus)	Medicines for diabetes should be easily available at government health facilities. Patients tend to shift towards private sector due to non-availability of medicines in government.
Home-delivered care	Home-delivered services	As is being done under TB program, program functionaries perceived that medicines for diabetes can also be home-delivered through village-level frontline health workers. This would reduce the clinic visits and thereby the costs incurred.
	Early diagnosis nearby	Early diagnosis at a place near the villages of the patients would help reduce costs incurred for transportation. It would also save their time and avoid loss of work.
Timely referral	Referral by private doctors	Patients perceived that if private doctors refer them early to the government hospitals, then they would not incur more expenses.
